# Unveiling Cacao Rootstock-Genotypes with Potential Use in the Mitigation of Cadmium Bioaccumulation

**DOI:** 10.3390/plants12162941

**Published:** 2023-08-14

**Authors:** Donald A. Galvis, Yeirme Y. Jaimes-Suárez, Jairo Rojas Molina, Rosalba Ruiz, Clara E. León-Moreno, Fabricio Eulalio Leite Carvalho

**Affiliations:** 1 Centro de Investigación La Suiza, Corporación Colombiana de Investigación Agropecuaria (Agrosavia), Rionegro 250047, Colombia; 2 Facultad de Ciencias Básicas, Universidad de Córdoba, Montería 230002, Colombia; rruizv@correo.unicordoba.edu.co

**Keywords:** rootstock, heavy metals, cadmium toxicity, phytoremediation, phytostabilizing, *Theobroma cacao*

## Abstract

The accumulation of high cadmium (Cd) levels in cacao beans (*Theobroma cacao*) generate several commercial and health issues. We hypothesized that cacao phenotypic and genotypic diversity could provide new insights to decrease Cd accumulation in cacao beans. Nine cacao rootstock genotypes were evaluated for up to 90 days under 0, 6, and 12 (mg·kg^−1^) of CdCl_2_ exposure and Cd content and plant growth dynamics were measured in leaves, stems, and roots. Data revealed that all cacao genotypes studied here were highly tolerant to Cd, since they presented tolerance index ≥ 60%. In shoots, EET61 and PA46 presented the higher (~270 mg·kg DW^−1^) and lower (~20 mg·kg DW^−1^) Cd concentration, respectively. Accordingly, only the EET61 showed an increase in the shoot cadmium translocation factor over the 90 days of exposure. However, when analyzing cadmium allocation to different organs based on total plant dry mass production, none of the genotypes maintained high Cd compartmentalization into roots, since P46, which was the genotype with the highest allocation of Cd to the roots, presented only 20% of total cadmium per plant in this plant organ and 80% allocated into the shoots, under Cd 12 (mg·kg^−1^) and after 90 days of exposure. Thus, genotypic/phenotypic variability in cacao rootstocks may provide valuable strategies for maximizing the reduction in Cd content in shoots. In this sense, IMC67 and PA46 were the ones that stood out in the present study.

## 1. Introduction

Cacao (*Theobroma cacao* L.) is a tree species of the Malvaceae family that originated in Amazonia. The use of almonds obtained from the cacao fruit as raw material for making chocolate refers to an ancient custom among the natives of this region of South America [1]. Countries like Colombia show a positive economic balance related to the crop, increasing exports by up to 13% in the last five years [2]. This behavior in the market reflects the importance gained at a regional level. Countries such as Brazil, Colombia, Ecuador, Peru, and Venezuela stand out in cacao production [3], highlighting that Latin America produces 80% of the world’s “prime” cacao. Indeed, 70% of the region’s total cacao exports correspond to fine varieties of cacao, which could be considered a consequence of its genetic diversity.

Cacao demands strict quality and safety requirements, and the concentration of cadmium (Cd) is one of the most relevant parameters for its commercialization. Indeed, an intimate relationship between the crop and this metal has been observed, demonstrating a greater predisposition to Cd accumulation in cacao. As a matter of fact, the finished chocolate may present higher cadmium concentration than other foods and beverages [4]. Several problems in human health are associated with Cd, ranging from injuries during pregnancy, gastrointestinal irritation, nausea, vomiting, kidney damage, emphysema, and lung cancer [5,6]. For this reason, the European Union (EU) established limits ranging between 0.10 and 0.80 mg Cd kg^−1^ of dry matter according to the percentage of crude cacao present in the final product [7].

Additionally, excess Cd may lead to oxidative stress in the plants due to its pro-oxidant activity, affecting plant growth and productivity [8]. In addition, Cd may replace essential cations (Ca^2+^, Fe^2+^, Mg^2+^, and Zn^2+^) in crucial enzymes leading to its loss of function [9,10,11]. So, to counter this problem, Cd tolerance mechanisms have been reported in plants and bacteria. These mechanisms encompass the exclusion of Cd, its active excretion, restricted translocation to strategic organs, redistribution to less crucial tissues, and chelation and compartmentalization in vacuoles [12,13,14]. These capabilities can be affected by the environment but largely depend on the genetic background of each species or variety.

Previous studies evidenced the broad phenotypic plasticity related to heavy metal responses in cacao. For instance, the response to the strain induced by Pb is broadly different among genotypes [15]. In Peru, Arévalo-Gardini et al. [16] reported a greater predisposition to the accumulation of Cd in the CCN51, ICS95, and some hybrids compared to the native genotypes. Lewis et al. [17] also reported a variation of up to 13 times in the concentration of Cd in cacao beans among the 100 genotypes evaluated. In the same context, Engbersen et al. [18] reported that the POUND7 genotype presented a lower predisposition to incorporate Cd in the grain, differing significantly from the other ten cultivars evaluated. Similarly, evidence suggests that cacao beans have different concentrations of Cd depending not only on the variety but also on the geographical location. Indeed, the concentration of Cd in South America is almost three times higher than in Central America and East Africa and ten times higher than that of West Africa [19].

The situation in Colombia is not unrelated to that reported in the South America region, raising concern about the future of cacao exports and its derivatives. In analyses carried out for the recognition of cacao beans in crops located in the department of Santander, Colombia, 57% of the samples collected presented levels that exceeded the maximums established by the European Community [20]. In the same way, Aguirre-Forero et al. [21] reported that in the Magdalena departmental region, the content of Cd in the cacao samples also exceeded the permissible limits by the EU. Bravo et al. [22] reported that about 42% of the samples taken from the main cacao-producer departments in Colombia exceeded the threshold defined for the natural concentration of Cd in soil established by the Finnish Ministry of the Environment [23]. Therefore, there is an urgent demand for new techniques to mitigate the problem caused by excess Cd in cacao.

The use of asexual propagation employing grafting to achieve a higher precocity, uniformity, quality, and productivity in cacao is strongly recommendable [24]. Thus, the use of genotypes with low Cd accumulation/translocation as rootstock plants could be a possible alternative for mitigating Cd bioaccumulation in cacao. This hypothesis suggests a potential for taking advantage of the intrinsic genetic variety of cacao genotypes to obtain a more effective design for Cd exclusion from the shoots. However, to the best of our knowledge, research on this topic is still limited in the literature [25]. For this reason, in the present study, Cd bioaccumulation was evaluated in nine cacao genotypes under greenhouse conditions, selected as cultivars recommended for use as rootstocks in Colombia [26]. Moreover, all these nine genotypes previously demonstrated important agronomic characteristics, such as resistance to diseases produced by the pathogens *Ceratocystis* sp. and *Phytophthora* sp. [27,28], which must be considered as an upset. The dynamics of Cd bioaccumulation and allocation into different plant organs as a response to genotype-contamination interactions are discussed below.

## 2. Results

### 2.1. Rootstock Genotypes PA121, IMC60, and IMC67 Exhibited Better Growth in Cadmium-Contaminated Substrates

For the present study, a growth analysis was performed to verify the impact of different cadmium chloride concentrations on the physiological response of the selected cacao rootstock genotypes. Non-destructive growth variables (plant height, stem diameter, and the number of leaves) were determined from 30 days before Cd application, every 30 days until 90 days of exposure. None of these variables showed significant changes in response to Cd treatment, despite the different genotypes evaluated (Figures S1–S3). On the other hand, destructive growth variables (leaf dry mass, stem dry mass, root dry mass, and shoot dry mass) were recorded every 30 days, starting with a sampling after the first 30 days of Cd exposure.

**Figure 1:**
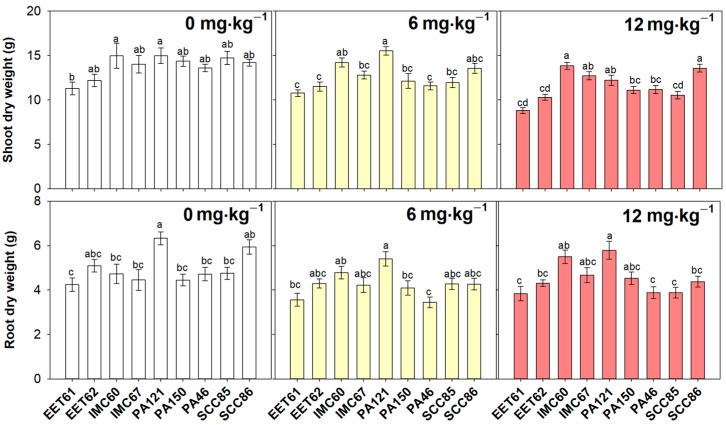
Dry mass in roots and shoots of nine cacao rootstock genotypes exposed to different levels (0, 6 and 12 mg·kg^−1^) of cadmium (CdCl_2_) for 90 days. Bars represent average ± standard error (*n* = 9). Different letters show significant differences between cacao genotypes in the same cadmium treatment, according to Tukey’s test (*p* < 0.05).

After 90 days of exposure to 6 mg·kg^−1^ Cd, the PA121 genotype exhibited the highest shoot dry mass, presenting a significant increase in comparison to the EET61, EET62, PA46, PA150, SCC85, and IMC67 genotypes. However, after 90 days of 12 mg·kg^−1^ Cd exposure, shoot dry mass was greater in IMC60 e SCC86, with significant differences to EET61, EET62, PA46, PA150, and SCC85 (Figure 1). Indeed, the genotypes EET61, EET62, PA150, PA46, and PA121 showed significant decreases in leaf dry mass under 12 mg·kg^−1^ of Cd, when compared to their respective controls, while SCC85 exhibited a decrease in leaf dry mass in both Cd treatments (Table S2). Also, regarding the leaf dry mass, the IMC60 and SCC86 genotypes did not show significant effects in response to any of the Cd treatments, thus corroborating the improved growth of these genotypes under Cd exposure. The EET62, IMC60, IMC67, and PA121 genotypes, in turn, did not show significant changes in stem dry mass in response to Cd (Table S2), suggesting a lower susceptibility of cacao stem tissues to stress caused by Cd. Regarding the root dry mass, PA121 also exhibited the highest performance under 6 mg·kg^−1^ of Cd supply, presenting a significant increase in mass as compared to EET61, PA46, and PA150 (Figure 1). Additionally, under 12 mg·kg^−1^ of Cd stress, the cacao genotype PA121 also presented higher dry root mass, with a statistical difference compared to genotypes EET61, EET62, PA46, SCC85, and SCC86 (Figure 1). Taken together, the data showed evidence for better growth performance by the genotypes PA121, IMC60, and IMC67 under conditions of substrate contamination by CdCl_2_.

**Figure 2:**
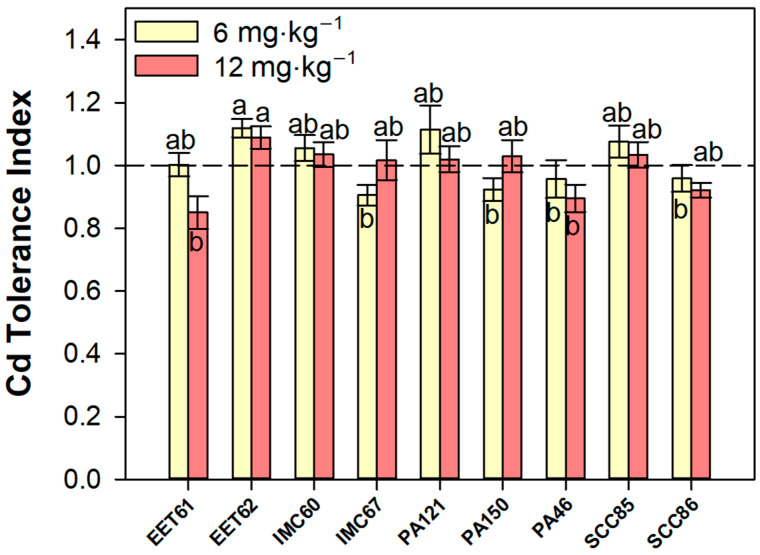
Cadmium tolerance index (TI) of nine cacao rootstock genotypes exposed to different levels (6 and 12 mg·kg^−1^) of CdCl_2_ for 90 days. Bars represent average ± standard error (*n* = 9). Different letters show significant differences between cacao genotypes according to Tukey’s test (*p* < 0.05).

When analyzing the differences in tolerance of the different cacao rootstocks, it is important to verify that EET61, for example, already showed lower growth compared to the highlighted PA121 genotype even under reference conditions (Figure 1). In fact, EET61 was the only genotype to show a statistical difference in the relative growth rate (RGR) under 12 mg·kg^−1^ of Cd in both time intervals analyzed: 30–60 and 60–90 days (Figure S4). To deepen the comparative tolerance analysis, the cadmium tolerance index was estimated based on the decrease in total dry mass presented in Cd-treated plants compared to the respective controls. The Cd tolerance index average estimated over 90 days of exposure evidenced EET62 as the most tolerant genotype to Cd (in both concentrations evaluated), showing a significant difference concerning the genotypes IMC67, PA150, PA46, and SCC86 in 6 mg·kg^−1^ of contamination (Figure 2). Under 12 mg·kg^−1^ of Cd exposure, only the EET62 genotype was statistically more tolerant to cadmium as compared to PA46 (Figure 2).

### 2.2. PA46 and IMC67 Genotypes Exhibited Lower Cd Content in Shoots, Highlighting Their Potential Use for Cadmium Mitigation Strategies

**Figure 3:**
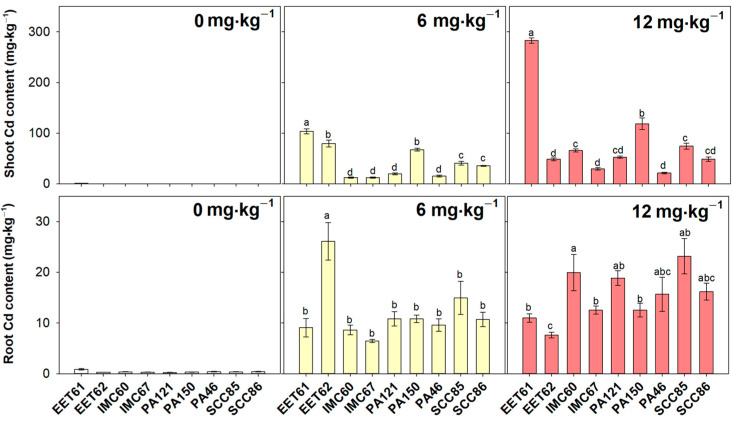
Cadmium content in roots and shoots of nine cacao rootstock genotypes exposed to different levels (0, 6 and 12 mg·kg^−1^) of cadmium (CdCl_2_) for 90 days. Bars represent average ± standard error (*n* = 3). Different letters show significant differences between cacao genotypes in the same cadmium treatment, according to Tukey’s test (*p* < 0.05).

Under control conditions, Cd content in leaves, stems, shoots, and roots was negligible and all genotypes were affected by the increase in Cd content, due to exposure to 6 or 12 mg·kg^−1^) CdCl_2_, as compared to the respective controls (Table S3). In shoots after 90 days of 6 mg·kg^−1^) CdCl^2^ exposure, PA46, IMC67, PA121, and IMC60 were the genotypes with lower Cd content, followed by SCC85 and SCC86; PA150 and EET62, and, at least, EET61 (Figure 3). Under 12 mg·kg^−1^, the Cd shoot content in PA46, IMC67, and EET62 was the lower among the studied genotypes, followed by SCC86, PA121, IMC60, and SCC85; PA150, and, at least, EET61 (Figure 3). These results especially highlight PA46 and IMC67 genotypes for having low concentrations of Cd in shoots, which may represent a very favorable characteristic to mitigate the accumulation of this metal in cacao. Regarding root Cd content, after 90 days of exposure to 6 mg·kg^−1^ CdCl_2_, EET62 was the most prominent Cd concentrator (Cd/tissue dry mass), exhibiting approximately double Cd content as compared to all other evaluated genotypes (Figure 3).

**Figure 4:**
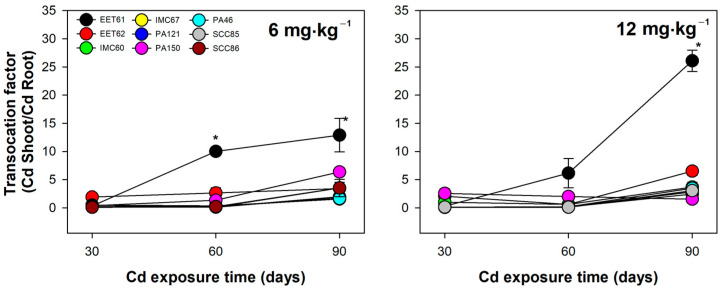
Translocation factor (TF) of cadmium of nine cacao rootstock genotypes exposed to increasing levels (6 and 12 mg·kg^−1^) of cadmium (CdCl_2_) for up to 90 days. Circles represent the mean and bars indicate the standard error of the mean (*n* = 3). Asterisks mean significant difference between cacao genotypes at the same time of exposure, according to Student’s *t* test (*p* ≤ 0.05).

Under 12 mg·kg^−1^ of Cd exposure, inversely, EET62 exhibited the lowest Cd content in roots, followed by EET61, IMC67, and PA150, and finally PA46, SCC86, SCC85, PA121, and IMC60 (Figure 3). Therefore, these results reinforce the existence of contrast in the levels of cadmium concentration in shoots of different genotypes of cacao rootstocks, which justifies its strategic use to achieve best results in terms of low Cd translocation to important organs such as fruits and grains. EET61 was the most prominent rootstock in the translocation of cadmium from roots to shoots (Figure 4), which coincide with the low tolerance of this genotype, especially under 12 mg·kg^−1^ of CdCl_2_ (Figure 2). Taken together, the results indicate that the concentration of cadmium in the different tissues of cacao rootstock genotypes depends not only on the genetic material per se but also on the intensity of exposure to the metal concentration in the soil solution. This observation implies that future mitigation strategies based on the use of contrasting genotypes should be designed in a dose-dependent way. 

### 2.3. Cadmium Allocation in Roots, Stem, and Leaves Suggests Different Physiological Strategies to Cope with Excess cd in Cacao Rootstock Genotypes

Comparing the content of cadmium among different plant tissues is a complex task, from a physiological point of view. The problem with doing this comparison freely is that the cadmium content presents a variable base rate. In plants, not only the amount of Cd can fluctuate as a function of time, but also the base of reference, i.e., the dry mass of the specific plant organ may also change over time, even because of the interaction with cadmium itself. Therefore, a more in-depth analysis of the responses of different cacao rootstock genotypes to CdCl_2_ contamination was performed here based on the quantitative allocation of cadmium in different plant tissues as a function of time.

**Figure 5:**
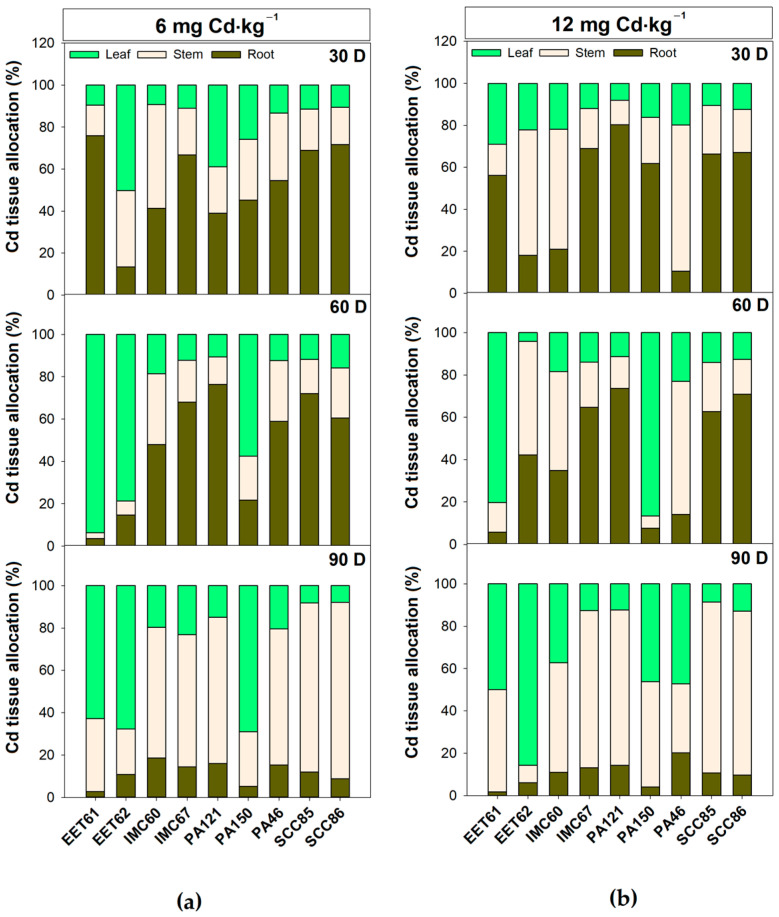
Cadmium allocation in different tissues (leaves, stems, and roots) in different genotypes of cacao rootstock exposed to (**a**) 6 mg·kg^−1^ CdCl_2_ (**b**) 12 mg·kg^−1^ CdCl_2_ contamination for up to 90 days. Data are presented based on the proportion of Cd content determined (mg·kg^−1^) adjusted by the accumulated dry weight (g) per plant organ at each sampling point. Results are expressed in mg cadmium per plant organ (leaf, stem, and roots).

In the first 30 days of exposure to 12 (mg·kg^−1^) CdCl_2_, most cacao genotypes, except for PA46, EET62, and IMC60, showed a higher amount of Cd allocated to root tissues (Figure 5a). This allocation, however, became predominantly directed to shoots at 90 days of exposure in all the evaluated genotypes, with emphasis on EET61, which presented 98% of the cadmium allocated between leaf and stem tissues. Interestingly, when the exposure dose to cadmium was equal to 6 (mg·kg^−1^), the capacity to allocate cadmium to root tissue was more prevailing. This tendency of Cd allocation to roots was observed up to 60 days in the genotypes IMC60, IMC67, PA121, PA46, SCC85, and SCC86 (Figure 5b). These data suggest that the cadmium accumulation strategy in specific tissues may not only be genotype-dependent but also dose- and time-dependent, which add greater complexity to the selection of materials with the specific purpose of mitigating Cd contamination in cacao grains.

Additionally, it is important to highlight here that the total amount of Cd per plant strongly contrasts among the different genotypes evaluated. PA46 and IMC67 exhibited the lowest Cd accumulation after 90 days (0.30 and 0.44 mg plant ^−1^, respectively)—(Figure S5). Additionally, these genotypes showed a relative constancy in the amount of cadmium over time, suggesting stability or absence of Cd accumulation, which could suggest a possible exclusion or avoidance mechanism despite the high concentration in the substrate (Figure S5). Similarly, an interesting response was presented by EET62, which showed a reduction in the amount of Cd per plant as a function of time. The other genotypes all showed a tendency to accumulate cadmium in plants as a function of time, which is an energetically favorable flux because of the high concentrations of cadmium in the external root medium. 

EET61 showed the highest accumulation of cadmium per plant (2.53 mg plant^−1^), with 50% allocated to leaves (Figure 5 and Figure S5). PA121 also showed a tendency to accumulate Cd over time (reaching 0.75 mg plant^−1^ after 90 days—Figure S5). Therefore, based on these results, it can be assumed that upon exposure to 12 mg·kg^−1^ Cd, all cacao genotypes showed an initial tendency to allocate this metal to the roots that lasts at least up to 30 days. After this period, a tendency of allocation to shoot tissues prevailed.

## 3. Discussion

The data compiled in the present study reinforce the possibility of using cacao rootstock genotypes specifically selected for their ability to mitigate cadmium accumulation in fruits and beans. It is important to stress here that, in the present study, the evaluated cacao plants were in the juvenile stage; also, analyses of cadmium content directly in fruits and grains were not carried out. However, in the literature, a highly positive correlation was thought between the cadmium content in the shoot and the content effectively accumulated in fruits [17]. Indeed, once Cd is loaded into the xylem, there is a high probability that the metal reaches the fruits as well as may be translocated to the leaves and fruits. Despite these important caveats, the results obtained here strongly contribute to the prospection of different cacao materials that may be deeply investigated in the future, preferably in adult plants under field conditions. 

In this primary prospect, it is also important to highlight that the genotypes analyzed here may present marked differences in physiological strategy and, consequently, in growth performance and Cd translocation, which depends directly on the level of substrate contamination. With this emphasis, aiming for substrates with contamination levels close to 6 mg Cd kg^−1^_,_ the following order of genotype ranking is recommended based on their potential for mitigating Cd translocation to shoots: PA46, IMC67, IMC60, PA121, SCC86, SCC85, PA150, EET62, and, finally, EET61. On the other hand, under higher contamination conditions (~12 mg Cd kg^−1^), the following ranking was considered: PA46, IMC67, EET62, SCC86, PA121, IMC60, SCC85, PA150, and EET61.

Cacao is a heterozygous cross-fertilization plant, which can greatly increase its potential for genetic variability. In this context, the use of grafting techniques in cacao has several advantages from a productive point of view and, above all, because it allows the selection of favorable characteristics against adverse conditions. In general, for the selection of suitable rootstocks, characteristics such as: influence on early fruiting are considered; transmission of vegetative vigor to the canopy, acclimatization to the medium, tolerance to adverse biotic and abiotic factors, longevity, and, above all, its effect on crop variability [26]. These characteristics contrast with those selected in scion genotypes that generally include high productivity and specific quality aspects. 

For the specific case of the rootstock genotypes used in the present study, they previously demonstrated important agronomic characteristics, such as resistance to diseases produced by the pathogens *Ceratocystis* sp. and *Phytophthora* sp. [27,28], which must be considered as an upset. Additionally, in the case of genotypes PA121, PA46, and IMC67, previous results showed their relative tolerance against excess aluminum in soils [26]. Therefore, our results are consistent with these previous findings that point to the beneficial potential, especially of the P46 and IMC67 genotypes in cacao.

Interestingly, even at contamination levels as high as 12 mg·kg^−1^ of cadmium, all evaluated genotypes presented a relatively high resistance to cadmium stress, since the lowest “tolerance index” values obtained were around 0.85 (EET61 at 12 mg·kg^−1^), which is a very high value. According to the classification proposed by Lux et al. [29], all the genotypes evaluated would be defined as highly “tolerant” (TI ≥ 60%). Therefore, it can be concluded that during the performed trial, no cacao rootstock genotype was found under cadmium physiological stress. It is more probable that plastic or elastic responses associated with plant acclimatization against the Cd-induced strain (see Blum [30] for strain definition) were triggered in all the evaluated genotypes. An interesting aspect can also be highlighted from the results obtained here: EET61, the genotype presenting higher cadmium accumulation in shoots, and PA46, the genotype with lower Cd accumulation, both showed high acclimation to Cd, based on the TI values close to 0.85. The physiological strategies related to this similar TI degree, however, must have been very distinct, as corroborated by the different Cd contents and allocation found in these plants. 

The behavior shown by genotypes such as EET61, which presented a progressive accumulation of cadmium per plant (under 12 mg·kg^−1^), can suggest an effective acclimation mechanism against Cd stress. It can be considered that the acclimation mechanism was effective because within the time frame in which the plants were evaluated, there was no significant difference in plant height and number of leaves and the plant TI was higher than 60% [29]. Therefore, this genotype can grow under high concentrations of Cd, promoting its accumulation in plant tissues (mainly shoots), which is a common characteristic of physiological acclimation by tolerance strategies [30,31,32]. On the other hand, the PA46 also reached similar acclimation (TI = ~0.85 at 12 mg·kg^−1^ of Cd), but with a notably reduced concentration of Cd in shoots, and it can maintain very stable levels of total Cd accumulated per plant after 90 days of exposure. This type of plant response could evoke an avoidance strategy, which needs to be studied in more depth [30,31,32].

The tolerance to excess Cd in cacao plants could theoretically be reached by two very distinct strategies: (1) tolerance via compartmentalization of Cd in the roots or (2) tolerance via translocation of Cd to the shoots for posterior compartmentalization [33]. For cadmium mitigation purposes, only the first of these mechanisms would be of interest. However, the data compiled here revealed that any of these cacao genotypes can support long-term Cd root compartmentalization, especially under 12 mg·kg^−1^ contamination. In the case of 6 mg·kg^−1^ contaminated substrates, the potential of some genotypes (IMC60, IMC67, PA121, PA46, SCC85, and SCC86) to allocate Cd into roots persisted only until 60 days of exposure. In fact, when designing strategies to mitigate cadmium accumulation in crop shoots, it is important to focus on genotypes with higher Cd allocation in the roots or low Cd uptake overall. Thus, based on the data compiled here, Cd allocation into the roots may not represent the best reference trait for the cacao breeding programs focused on the Cd mitigation issue. 

There is no known metabolic function for Cd in plant cells; therefore, the energetic imbalance between available photochemical energy and its consumption in metabolism, as an indirect effect of excess Cd, is intrinsically associated with the accumulation of reactive oxygen species (ROS), which may promote cell death, chlorosis, and leaf senescence [34]. In addition, this negative response is commonly related to decreases in water and nutrients uptake, low rates of photosynthesis, and, consequently, stunted growth [35]. Consequently, these conditions associated with oxidative stress generated by excess cadmium can generate an energetic competition between the defense metabolism and the metabolism directed to growth and productivity, thus decreasing yield in non-tolerant crops. Indeed, to accumulate and tolerate high Cd concentrations in plant cells, the most common mechanism triggered by plant species consists of inducing the bioproduction of phytochelatins for vacuole compartmentalization of Cd, in a pathway derived from glutathione metabolism [36,37], which is also an important sink of ATP, NADPH, C, N, and S. 

On the other hand, to restrict cadmium absorbance cacao plants must probably go through the secretion of substances that promote the immobilization of Cd in the substrate [38] or the downregulation of metal transporters expression, such as NRAMP and HMA family [39], which also implies highly metabolic costs to the plant. Thus, similar to what may occur with other substances having high cytotoxic potential, like Na^+^ and NH_4_^+^ [40], under cadmium exposure, the most extreme defense mechanisms have high energetic costs, which may be ultimately reflected in plant growth. This phenomenon could explain why PA46 genotypes exhibited a higher decrease in growth despite lower Cd accumulation.

In practical terms, a shred of broad evidence on the influence of the rootstock on the accumulation of Cd in other species such as *Solanum photeinocarpum* [41], *Cyphomandra betacea* [42], *Galinsoga parviflora* [43], *Arabidopsis thaliana* [44], and *Glycine max* [45] is reported in the literature. Thus, the use of genotypes with low accumulation as rootstocks in commercial plantations could constitute a viable strategy for reducing the bioaccumulation of Cd in cacao beans in the short term. In cacao, Lewis et al. [17] reported the most extensive work carried out to date on different genotypes concerning the bioaccumulation of Cd, where they evaluated one hundred accessions distributed in eight genetic groups, according to Motamayor et al. [46]. This previous study revealed a similar Cd concentration for the IMC67 genotype, which can be classified among the 10 genotypes with the lowest accumulation of cadmium in this study. 

Interestingly, Engbersen et al. [18] studying the bioaccumulation of Cd in different genotypes, registered a higher concentration of Cd than those of the present study for the IMC67 genotype. These results are in accordance with the data obtained here and corroborate the potential of IMC67 rootstock for Cd mitigation strategies. Chupillon et al. [16] and Arevalo-Hernandez et al. [47] also evidenced a low Cd accumulation in the IMC67 shoots and, in turn, revealed high concentrations in the EET400 genotype. This response is consistent with the hyperaccumulating condition of the EET collection, also observed here, especially in EET61. Barraza et al. [48] also reported the highest concentrations of Cd in the leaves of EET103, EET116, and EET576 genotypes. To the best of our knowledge, this is the first study that highlighted the PA46 genotype as a potential mitigator of Cd accumulation, which has a TI like the EET61 genotype (~0.85) but probably exhibits stress resistance through a distinct Cd uptake avoidance mechanism.

More recently, another PA collection genotype, PA121 (also highlighted here at 6 mg·kg^−1^ contamination levels), was investigated in parallel to IMC67 under high Cd exposure conditions [25]. The authors screened for physiological and Cd tolerance markers in controlled crosses between the reference genotypes IMC67 and PA121, which were used as rootstocks in grafts with ICS95 and CCN51. The authors employed in these experiments Cd-spiked soil mixes with final Cd content of 7.49 mg·kg^−1^. The authors also reported increased Cd content in roots of PA121 as compared to IMC67, but similar levels in the leaves, which corroborates the data presented here. In addition, under such Cd conditions (7.49 mg·kg^−1^), PA121 genotype exhibited more intense photoinhibition as compared to IMC67, which was probably related to increased PSII photodamage [25].

These previous reports reinforce the hypothesis raised in the present study that effects associated with energy imbalance as a consequence of the activation of different physiological strategies of resistance to Cd in cacao are probably more restrictive than the direct cytotoxic effect generated by Cd *per se*, as evidenced by the differences between the IMC67 and PA121 genotypes, which have similar leaf Cd levels but strong contrasts related to photosynthetic metabolism [25]. Further studies on the limiting energetic-metabolic effects associated with exposure to Cd in different cacao rootstock genotypes are still needed.

Taken together, the data obtained here suggest that cacao rootstock genotypes exhibit important physiological characteristics to be exploited as rootstocks and promote Cd mitigation cultivation systems. Despite Cd compartmentalization in the roots not being a strategy sustained in the long term by the evaluated plants, some genotypes are capable of preventing cadmium accumulation at the whole plant level by restricting its uptake. Thus, based on the overall results, PA46 and IMC67 genotypes are the most promising for further breeding programs. Nevertheless, EET62 is also considered promising, especially to very high-Cd-contaminated areas since these plants exhibit low Cd overall uptake and higher tolerance index.

## 4. Materials and Methods

### 4.1. Experimental Location

The plants were grown under greenhouse conditions at the Agrosavia “La Suiza” Research Center located in the municipality of Rionegro, department of Santander (7°22′12″ N) (73°10′39″ W). Located at 530 m above sea level under natural sunlight at an average temperature of 25 ± 4 °C and an average photosynthetic photon flux density of 1390 ± 534 μmol m^−2^ s^−1^ at noon.

### 4.2. Establishment of Experiments

Seeds of the EET61, EET62, IMC60, IMC67, PA121, PA150, PA46, SCC85, and SCC86 genotypes from the Germplasm Bank of the La Suiza Research Center were used. These seeds were pre-germinated in sphagnum peat and later transplanted into the substrate. The plants were grown in polyethylene pots with a volume of 2.8 L. The substrate consisted of soil (first 30 cm), fine river sand, and organic compost (3:1:0.5). This substrate was crushed, sieved, and disinfected using the solarization technique with a black plastic cover. The experiment was subjected to 65% shade conditions by nursery mesh. Daily irrigations were carried out, and weed control was performed manually. The chemical properties of the substrate used are summarized in Table S1. Cadmium chloride (CdCl_2_—SIGMA-ALDRICH, Cadmium chloride-99.99% trace metals basis) was used as the source of cadmium. The supplying of Cd (6 and 12 mg·kg^−1^) was carried out 90 days after sowing (das), in the time 0 of exposure. The contamination was performed considering the total weight of the substrate. Then, 20 mL of CdCl^2^ solutions (adjusted for a final concentration of 6 and 12 mg·kg^−1^, considering the total weight of the substrate) were applied, respectively, with syringes (25 mL) in the substrate at four different and symmetrical holes around the plant, distancing each one about 5 cm from the central plant axis and reaching about 10 cm depth in the substrate. 

### 4.3. Growth Variables and Cadmium Content

Physiological growth parameters (plant height, basal diameter, and the number of leaves or leaflets) were recorded at 60, 90, 120, 150, and 180 days after sowing (das). In addition, three destructive samplings were carried out at 120, 150, and 180 das (30, 60, and 90 days after cadmium exposure). Growth parameters and the metal concentration accumulated in roots, stems, and leaves were evaluated in these 3 destructive samplings. For each studied plant, roots, shoots, and leaves were subjected to oven drying (60 °C) during 48 h and, subsequently, the dry mass was determined. The relative growth rate (RGR) was calculated following the equation proposed by Hunt [49], RGR = [ln (W2) − ln (W1)]/(t2 − t1); where W1 and W2 are the initial and final dry mass (g) in the time intervals of the samples t1 and t2. Therefore, in this study, two RGR intervals were quantified, which represented the differences between 60–30 and 90–60 days after exposure to the excess cadmium.

Determination of cadmium was performed by atomic absorption spectrophotometry using nitric-perchloric acid (3:1, *v/v*) digests [35]. The cadmium translocation factor [TF = Shoot Cd Content (mg·kg^−1^)/Root Cd content (mg·kg^−1^)] was calculated according to Zayed et al. [50]. In addition, the methodology proposed by Wilkins [51] was followed to calculate the tolerance index [TI = Cd (6 or 12 mg·kg^−1^) exposed whole plant dry weight/control whole plant dry weight × 100]. The TI was estimated at each destructive sampling (30, 60, and 90 days after exposure) and an average for the entire period was calculated for each genotype under each Cd exposure level (6 and 12 mg·kg^−1^).

### 4.4. Experimental Design and Statistical Analysis

The experiment was arranged under a completely randomized block design, consisting of a factorial 9 × 3 × 3 (9 cacao genotypes, 3 cadmium levels—0, 6, and 12 (mg·kg^−1^), and 3 times of destructive harvesting—30, 60, and 90 days of exposure). The experimental unity encompassed 20 independent plants, from which nine independent replicates were selected randomly and used per treatment (*n* = 9). In the case of cadmium determination, for each plant organ (root, stem, and leaves), a composing sample was prepared, which combined the 9 previous replicates in 3. Thus, for the specific case of cadmium determination, *n* = 3. The averages were calculated for each variable, and an analysis of variance on ranks (*p* ≤ 0.05) was performed. Significant differences detected by ANOVA were subsequently analyzed by a Tukey comparison test (*p* < 0.05) or Student’s *t*-test, as described in figure and table captions.

## Supplementary Materials

The following supporting information can be downloaded at: https://www.mdpi.com/article/10.3390/plants12162941/s1. Table S1. Chemical features of the substrate employed in the research. Table S2. Leaf, stem, shoot, and root dry weight (g) in different cacao rootstock genotypes. Asterisks represent significant differences according to Student’s *t*-test. Table S3. Cd content (mg·kg^−1^) in different cacao rootstock genotypes. Asterisks represent significant differences according to Student’s T-test (*, *p* < 0.05; **, *p* < 0.01) as comparing each Cd treatment with the control within the same exposure time. Figure S1. Height growth curves (cm) in different genotypes of cacao rootstock exposed to different doses of CdCl_2_. Figure S2. Stem diameter growth curves (mm) in different genotypes of cacao rootstock exposed to different doses of CdCl_2_ contamination (0, 6, and 12 mg·kg^−1^) for up to 90 days. Circles represent the average and bars indicate the standard error of the mean (*n* = 9). Figure S3. The number of leaves-based growth curves (n) in different genotypes of cacao rootstock exposed to different doses of CdCl_2_ contamination (0, 6, and 12 mg·kg^−1^) for up to 90 days. Circles represent the average and bars indicate the standard error of the mean (*n* = 9) of the mean (*n* = 9). Figure S4. Relative growth rate (RGR) of nine cacao rootstock genotypes exposed to different levels (6 and 12 mg·kg^−1^) of cadmium (CdCl_2_) for up to 90 days. The mean values with asterisks * show significant differences according to Tukey’s test (*p* < 0.05). Figure S5. Total cadmium accumulated per plant in different genotypes of cacao rootstock over time. Values of total cadmium accumulated per plant are obtained from the sum of the accumulated values in each part of the plant (leaves, stems, and roots). The cadmium values allocated to each plant part are obtained through the relationship between concentration (mg·kg^−1^) and dry mass (Kg DW) of each plant part, respectively. Circles represent mean (*n* = 3), and bars represent standard error of mean (SEM).
