# Supplementary material for: Unveiling Cacao Rootstock-Genotypes with Potential Use in the Mitigation of Cadmium Bioaccumulation
Source: Plants (Basel). 2023 Aug 14;12(16):2941. doi: 10.3390/plants12162941 (PMC10458809; doi:10.3390/plants12162941)
Supplement: Supplementary file 1 [file plants-12-02941-s001.zip › plants-2492900-supplementary.pdf]

Unveiling Cacao Rootstock-genotypes with Potential Use in the Mitigation of Cadmium Bioaccumulation

Donald A. Galvis, Yeirme Y. Jaimes-Suárez, Rosalba Ruiz, Jairo Rojas-Molina, Clara E. León-Moreno and Fabricio Eulalio Leite Carvalho

SUPPLEMENTARY MATERIAL

2023

Table S1. Chemical features of the substrate employed in the research.

Analytical determination	Value	Unit	Analytical determination	Value	Unit
Cadmium (Cd) **	0.64	$mg.kg^{-1}$	Acidity (Al+H) cmol(+)/kg KCl	N.D	cmol(+)/kg
pH (1:2.5)	6	pH units	(Al) Interchangeable	N.D	cmol(+)/kg
Electric conductivity (EC)	0.08	dS/m	Calcium (Ca) *	3.99	cmol(+)/kg
Organic material (OM) Walkey & Black	0.25	g/100g	Magnesium (Mg) *	0.35	cmol(+)/kg
Phosphorus (P) Bray II *	41.68	C	Potassium (K) *	0.1	cmol(+)/kg
Sulfur (S) *	2.61	$mg. kg^{-1}$	Iron (Fe) olsen *	37.41	$mg. kg^{-1}$
Effective cation exchange capacity (ECEC)	4.53	cmol(+)/kg	Copper (Cu) Olsen *	<1.0	$mg. kg^{-1}$
Boron (B) *	0.18	$mg. kg^{-1}$	Manganese (Mn) Olsen *	14.18	$mg. kg^{-1}$
Zinc (Zn) Olsen *	1.14	$mg. kg^{-1}$			

* Metal available values
** Pseudototal value

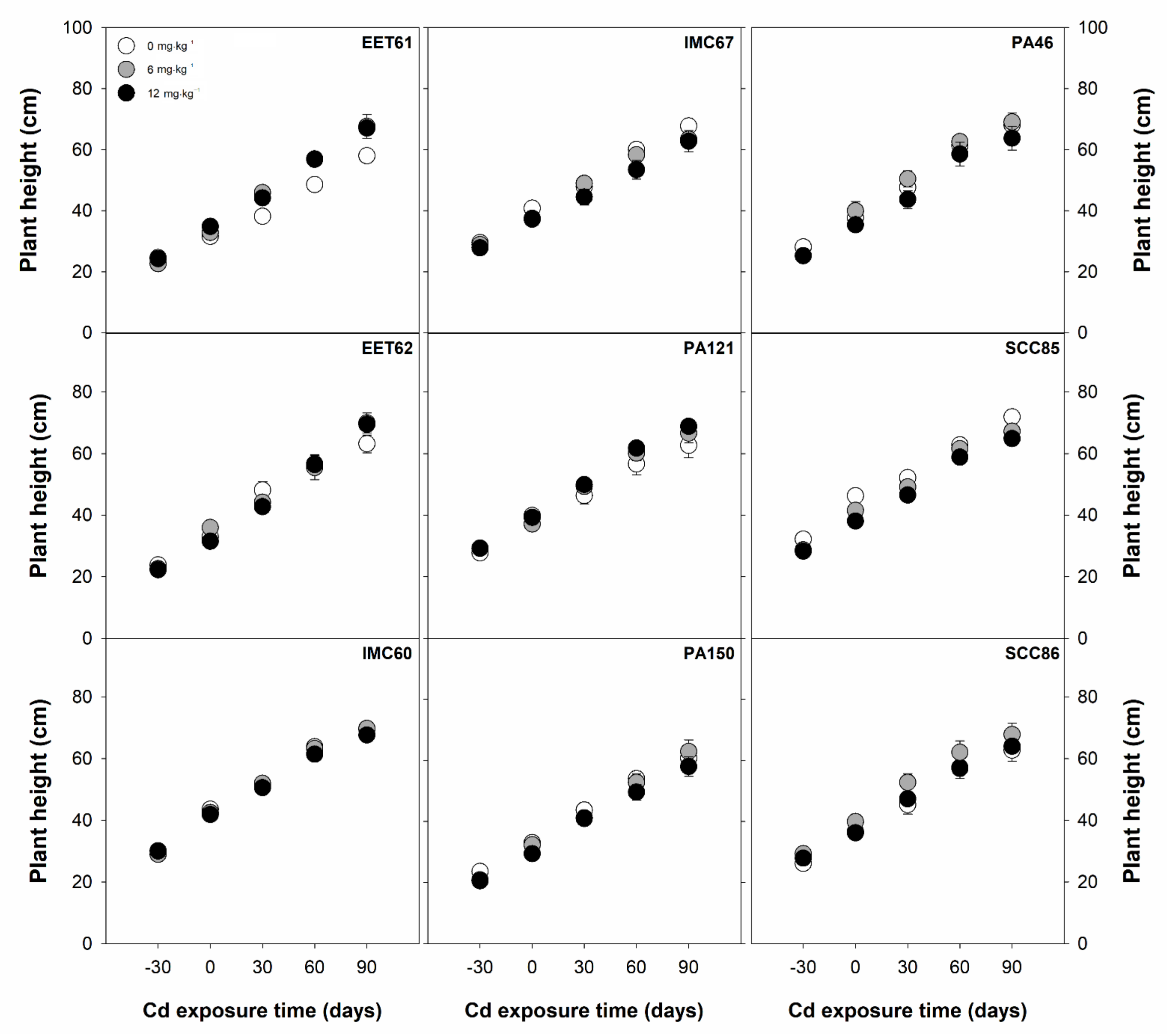

Figure S1. Height growth curves (cm) in different genotypes of cacao rootstock exposed to different doses of $CdCl_2$ contamination (0, 6, and 12 mg.kg^{-1}) for up to 90 days. Circles represent the average and bars indicate the standard error of the mean (*n* = 9).

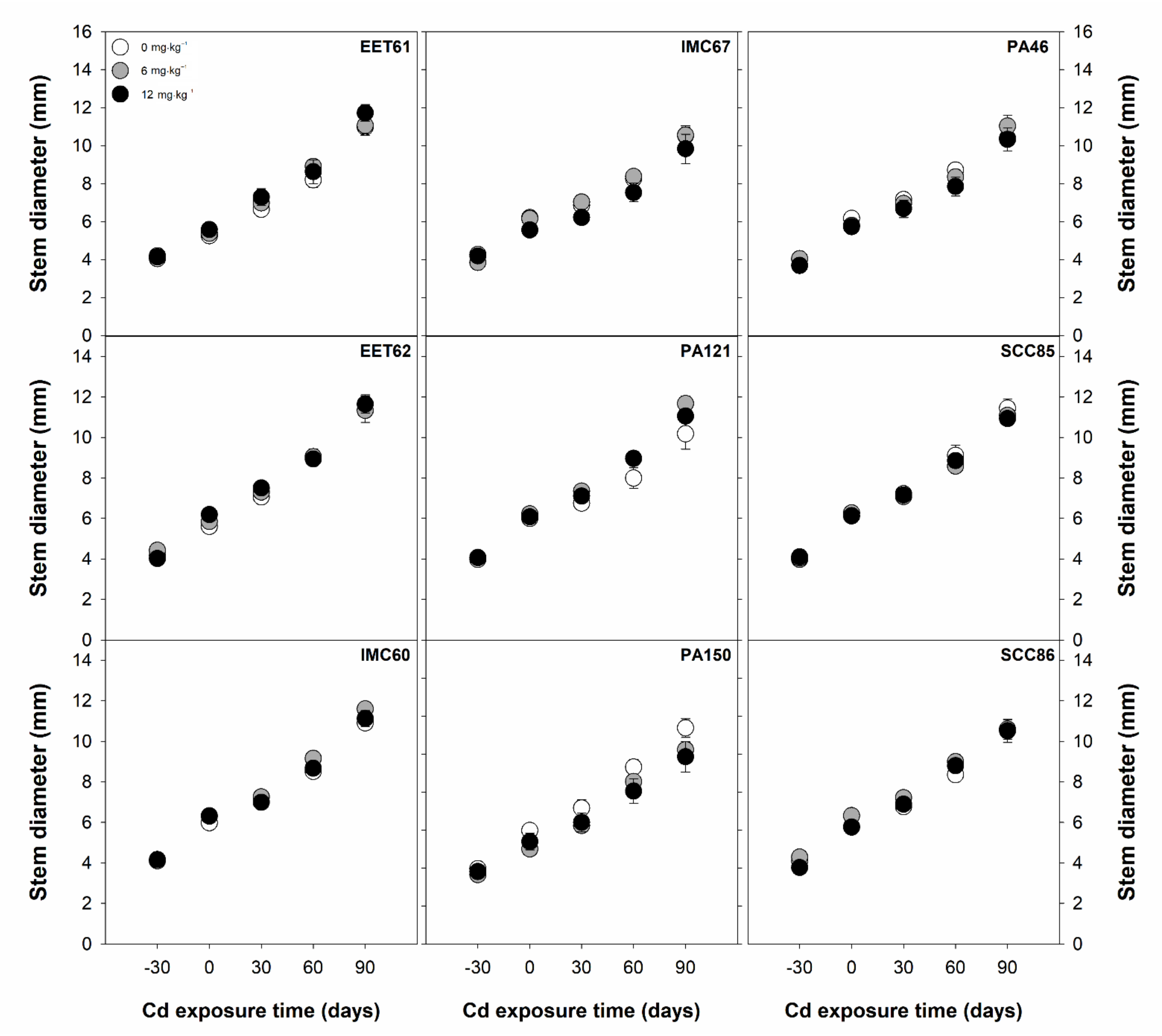

Figure S2. Stem diameter growth curves (mm) in different genotypes of cacao rootstock exposed to different doses of $CdCl_2$ contamination (0, 6, and 12 mg.kg^{-1}) for up to 90 days. Circles represent the average and bars indicate the standard error of the mean (*n* = 9).

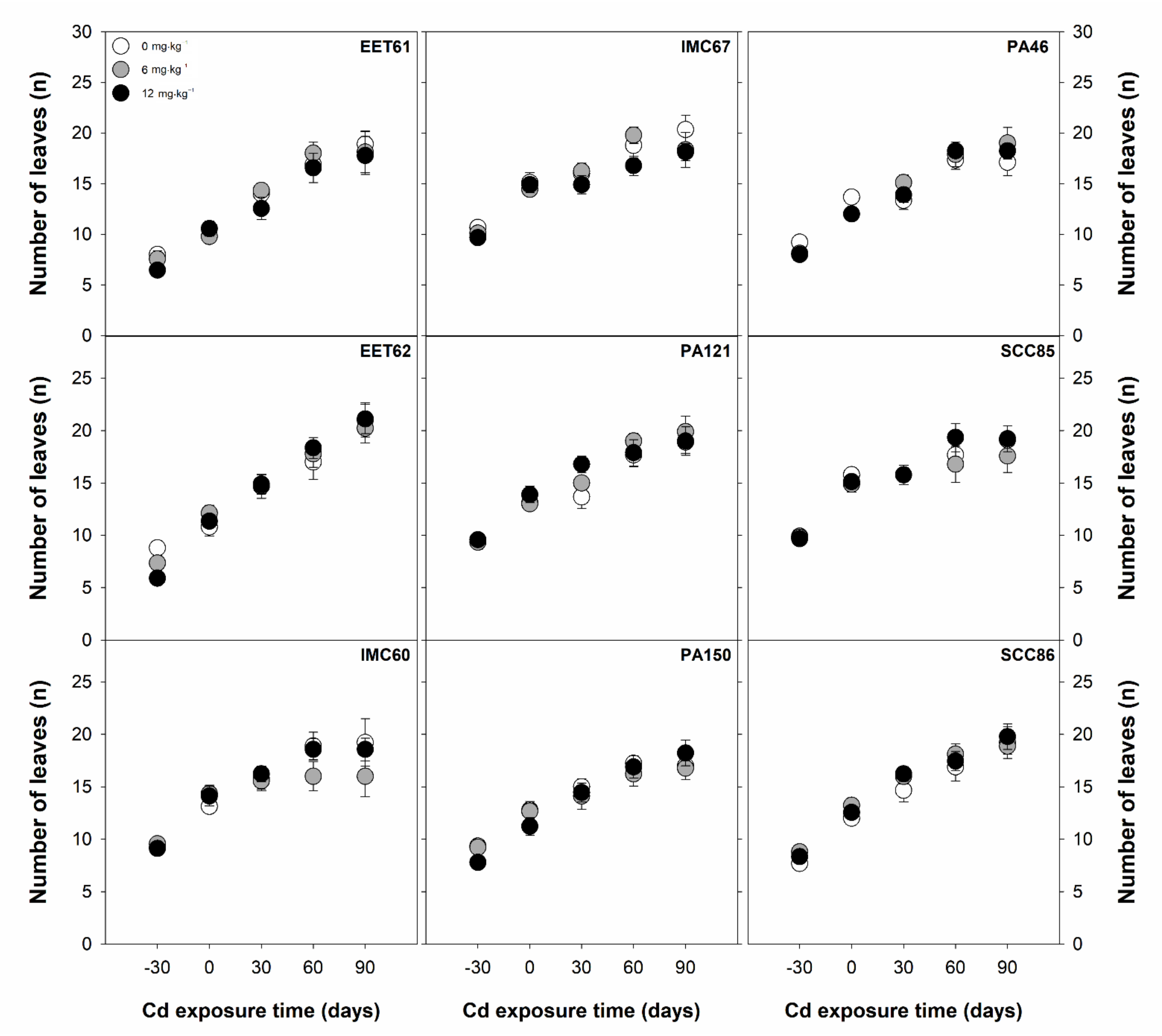

Figure S3. The number of leaves-based growth curves (n) in different genotypes of cacao rootstock exposed to different doses of $CdCl_2$ contamination (0, 6, and 12 mg.kg^{-1}) for up to 90 days. Circles represent the average and bars indicate the standard error of the mean (*n* = 9).

Table S2. Leaf, stem, shoot, and root dry weight (g) in different cacao rootstock genotypes. Asterisks represent significant differences according to Student's T-test (*, $p<0.05$; **, $p<0.01$)) as comparing each Cd treatment with the control within the same exposure time (*n*=3).

Genotype	Cd treatment	Time of exposure (days)	Leaf dry weight[a]			Stem dry weight[a]			Root dry weight[a]			Shoot dry weight[a]		
			AVR	SEM	*p*	AVR	SEM	*p*	AVR	SEM	*P*	AVR	SEM	*p*
EET61	0 mg.kg^{-1}	30	4,61	0,21		2,79	0,15		2,43	0,13		7,40	0,28	
		60	5,25	0,18		4,12	0,15		4,32	0,27		9,37	0,23	
		90	6,05	0,41		5,24	0,44		4,24	0,30		11,29	0,71	
	6 mg.kg^{-1}	30	4,28	0,19		3,36	0,20	*	3,20	0,15	**	7,64	0,30	
		60	4,80	0,37		4,69	0,37		3,31	0,37	*	9,49	0,59	
		90	5,28	0,32		5,50	0,21		3,56	0,29		10,78	0,37	
	12 mg.kg^{-1}	30	5,32	0,18	*	3,36	0,16	*	2,58	0,10		8,68	0,29	*
		60	6,04	0,27	*	3,73	0,17		2,98	0,13	**	9,78	0,39	
		90	4,94	0,23	**	3,86	0,12	*	3,83	0,33		8,80	0,33	**
EET62	0 mg.kg^{-1}	30	5,70	0,30		2,84	0,31		2,72	0,36		8,53	0,58	
		60	6,30	0,40		4,95	0,28		3,16	0,24		11,24	0,64	
		90	6,72	0,48		5,47	0,37		5,09	0,29		12,19	0,71	
	6 mg.kg^{-1}	30	5,42	0,24		3,62	0,18		2,72	0,19		9,05	0,33	
		60	5,47	0,37		5,06	0,26		3,96	0,26	**	10,53	0,54	
		90	6,09	0,36		5,43	0,21		4,29	0,21	*	11,52	0,52	
	12 mg.kg^{-1}	30	4,91	0,18	*	3,42	0,20		2,62	0,15		8,33	0,29	
		60	4,87	0,28	*	4,94	0,26		4,15	0,26	*	9,80	0,40	
		90	5,26	0,13	**	5,04	0,23		4,31	0,15	*	10,30	0,32	**
IMC60	0 mg.kg^{-1}	30	5,01	0,45		2,97	0,22		2,19	0,20		7,98	0,63	
		60	6,67	0,30		5,34	0,43		3,99	0,43		12,01	0,61	
		90	7,17	0,74		7,80	0,73		4,73	0,44		14,97	1,40	
	6 mg.kg^{-1}	30	5,39	0,36		3,25	0,15		2,29	0,21		8,64	0,41	
		60	6,15	0,41		5,03	0,20		3,31	0,23		11,18	0,55	
		90	6,72	0,30		7,50	0,36		4,79	0,28		14,22	0,51	
	12 mg.kg^{-1}	30	5,15	0,41		2,95	0,18		1,82	0,11		8,10	0,54	
		60	6,47	0,27		5,55	0,26		3,41	0,19		12,02	0,38	
		90	7,13	0,28		6,73	0,34		5,49	0,31		13,86	0,38	
IMC67	0 mg.kg^{-1}	30	5,43	0,40		2,80	0,25		1,89	0,17		8,22	0,59	
		60	7,13	0,38		4,57	0,37		3,30	0,27		11,70	0,72	
		90	7,16	0,51		6,87	0,61		4,45	0,47		14,03	1,00	
	6 mg.kg^{-1}	30	4,90	0,41		2,38	0,26		1,89	0,18		7,28	0,63	
		60	5,95	0,34	*	4,92	0,26		3,00	0,19		10,86	0,42	
		90	6,61	0,27		6,19	0,28		4,22	0,33		12,81	0,44	
	12 mg.kg^{-1}	30	5,41	0,38		3,14	0,32		2,82	0,43		8,56	0,60	
		60	6,52	0,48		4,66	0,29		3,20	0,30		11,18	0,71	
		90	6,94	0,35		5,77	0,23		4,67	0,34		12,71	0,45	
PA121	0 mg.kg^{-1}	30	5,03	0,37		3,04	0,13		2,47	0,17		8,06	0,46	
		60	6,84	0,41		5,15	0,39		3,57	0,25		12,00	0,73	
		90	6,88	0,27		8,11	0,73		6,33	0,29		14,99	0,88	
	6 mg.kg^{-1}	30	5,25	0,22		3,07	0,16		2,36	0,20		8,33	0,31	
		60	6,75	0,18		5,36	0,25		3,97	0,31		12,11	0,39	
		90	7,91	0,27	*	7,61	0,33		5,40	0,32	*	15,52	0,46	
	12 mg.kg^{-1}	30	5,46	0,33		2,87	0,16		2,71	0,25		8,34	0,37	
		60	5,68	0,34	*	4,95	0,40		3,26	0,35		10,64	0,63	
		90	5,81	0,35	*	6,40	0,41		5,79	0,40		12,21	0,57	*
PA150	0 mg.kg^{-1}	30	4,62	0,16		2,80	0,15		2,43	0,34		7,42	0,30	
		60	6,18	0,41		5,24	0,36		3,78	0,30		11,42	0,37	
		90	7,68	0,55		6,68	0,23		4,45	0,26		14,36	0,60	
	6 mg.kg^{-1}	30	5,05	0,23		3,51	0,28	*	2,33	0,12		8,55	0,38	*
		60	5,85	0,33		5,47	0,17		3,93	0,26		11,33	0,36	
		90	6,52	0,54		5,61	0,42	*	4,09	0,33		12,13	0,83	*
	12 mg.kg^{-1}	30	4,70	0,20		4,01	0,35	**	2,92	0,25		8,71	0,43	*
		60	6,32	0,38		5,16	0,24		3,47	0,42		11,48	0,39	
		90	5,72	0,18	*	5,40	0,29	**	4,53	0,28		11,12	0,41	**
PA46	Control	30	4,99	0,33		3,70	0,24		2,13	0,18		8,69	0,45	
		60	6,33	0,35		4,54	0,23		2,93	0,34		10,87	0,43	
		90	6,74	0,32		6,86	0,22		4,72	0,31		13,60	0,44	
	6 mg.Kg^{-1}	30	5,05	0,27		3,16	0,23		1,87	0,15		8,21	0,42	
		60	5,94	0,34		4,57	0,29		3,22	0,24		10,51	0,59	
		90	6,11	0,25		5,49	0,26	**	3,45	0,24	*	11,59	0,45	**
	12 mg.Kg^{-1}	30	5,01	0,44		3,33	0,20		2,28	0,14		8,34	0,62	
		60	5,34	0,33		4,00	0,26		2,75	0,31		9,34	0,42	*
		90	5,74	0,30	*	5,44	0,26	**	3,88	0,27	*	11,18	0,45	**
SCC85	0 mg.kg^{-1}	30	5,06	0,26		3,31	0,25		2,16	0,15		8,38	0,46	
		60	5,78	0,33		5,06	0,31		3,42	0,29		10,84	0,57	
		90	7,19	0,21		7,54	0,59		4,75	0,28		14,74	0,73	
	6 mg.kg^{-1}	30	4,61	0,49		3,00	0,17		2,09	0,11		7,61	0,51	
		60	6,38	0,28		5,09	0,27		3,52	0,19		11,47	0,50	
		90	5,97	0,36	*	5,99	0,31	*	4,28	0,26		11,96	0,58	**
	12 mg.kg^{-1}	30	5,02	0,24		2,90	0,10		1,72	0,07	*	7,92	0,32	
		60	5,69	0,20		4,93	0,18		3,57	0,35		10,63	0,34	
		90	5,17	0,24	**	5,37	0,27	**	3,88	0,24	*	10,54	0,42	**
SCC86	0 mg.kg^{-1}	30	5,67	0,26		2,88	0,14		2,18	0,10		8,55	0,25	
		60	6,71	0,24		5,16	0,29		3,39	0,28		11,87	0,30	
		90	6,70	0,31		7,48	0,25		5,93	0,32		14,18	0,36	
	6 mg.kg^{-1}	30	5,52	0,17		3,24	0,31		2,03	0,14		8,75	0,34	
		60	6,08	0,34		5,10	0,26		3,44	0,43		11,17	0,43	
		90	6,77	0,43		6,80	0,23	*	4,27	0,26	**	13,57	0,58	
	12 mg.kg^{-1}	30	5,09	0,21		2,83	0,18		2,09	0,10		7,93	0,34	
		60	6,49	0,23		4,48	0,15	*	2,99	0,10		10,97	0,21	*
		90	7,04	0,29		6,54	0,27	*	4,38	0,24	**	13,58	0,44	

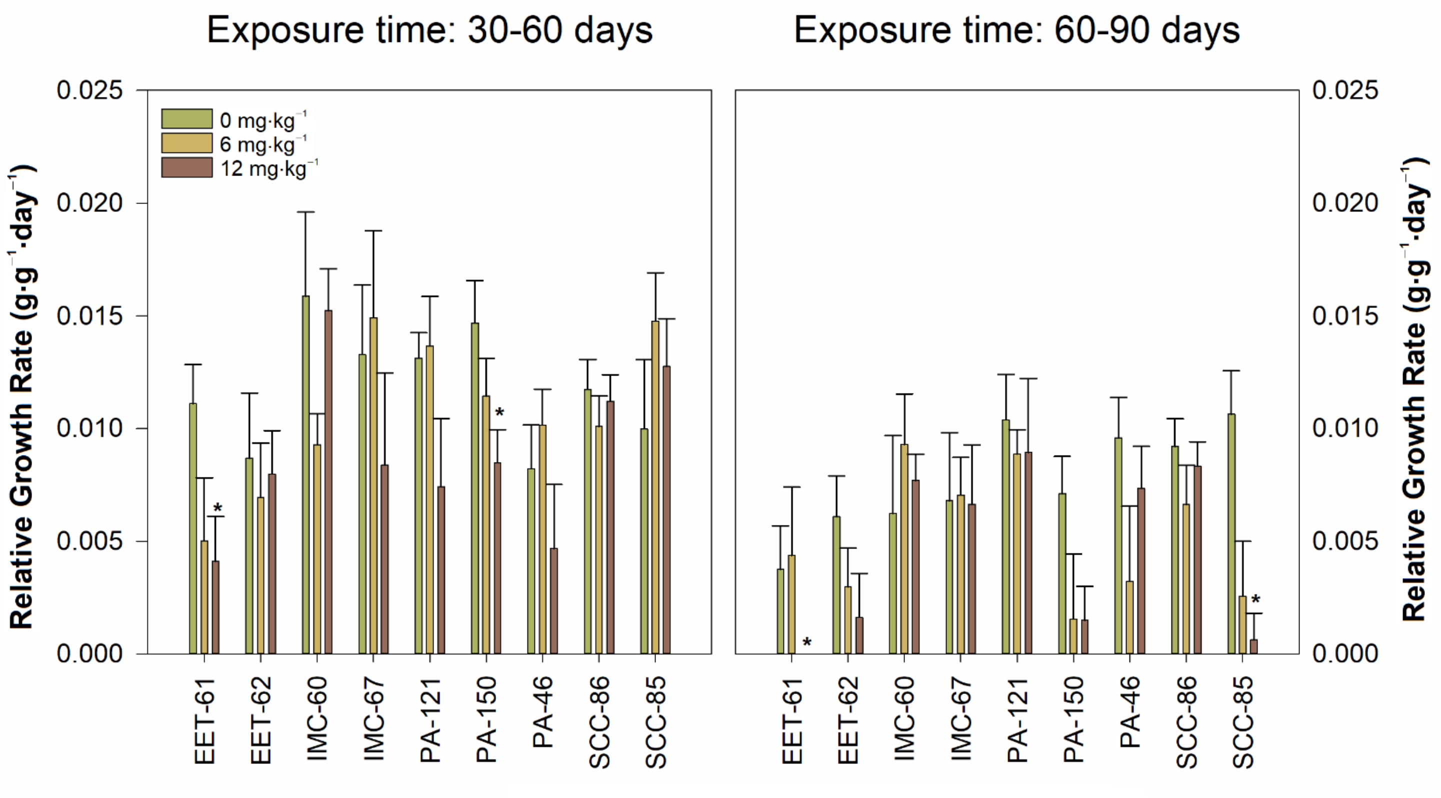

Figure S4. Relative growth rate (RGR) of nine cacao rootstock genotypes exposed to different levels (6 and 12 mg.kg^{-1}) of cadmium ($CdCl_2$) for up to 90 days. The mean values with asterisks* show significant differences according to Tukey's test ($p < 0.05$).

Table S3. Cd content (mg.kg^{-1}) in different cacao rootstock genotypes. Asterisks represent significant differences according to Student's T-test (*, p<0.05; **, p<0.01)) as comparing each Cd treatment with the control within the same exposure time.

Genotype	Cd treatment	Time of Exposure (days)	Leaf Cd content[a]			Stem Cd content[a]			Root Cd content[a]			Shoot Cd content[a]		
			AVR	SEM	*p*	AVR	SEM	*p*	AVR	SEM	*p*	AVR	SEM	*P*
		30	0,73	0,16		0,74	0,11		1,10	0,31		0,73	0,13	
	0 mg.kg^{-1}	60	0,25	0,02		0,33	0,02		0,20	0,03		0,28	0,02	
		90	1,93	0,07		0,33	0,06		0,85	0,14		1,19	0,09	
		30	1,95	0,40	*	3,92	1,24		8,65	1,16	*	2,66	0,24	*
EET61	6 mg.kg^{-1}	60	158,2	19,7	*	5,06	2,98		8,29	1,13	*	81,12	5,88	**
		90	136,75	4,72	**	72,87	4,51	**	9,07	1,81	*	103,85	4,95	**
		30	4,28	0,64		3,42	0,26		17,36	4,84		3,94	0,48	
	12 mg.kg^{-1}	60	86,87	18,1	*	24,43	4,30	*	12,20	2,17		62,24	9,14	*
		90	255,6	6,65	**	317,0	10,9	**	10,98	0,82		282,7	5,28	**
		30	0,62	0,19		0,74	0,13		1,19	0,31		0,67	0,16	
	0 mg.kg^{-1}	60	0,24	0,06		0,30	0,10		0,51	0,27		0,26	0,08	
		90	0,37	0,04		0,42	0,08		0,28	0,01		0,40	0,05	
		30	5,61	1,10	*	6,33	1,59		3,06	0,31	*	5,92	0,78	*
EET62	6 mg.kg^{-1}	60	188,2	28,0	*	16,70	2,32	*	48,00	10,3	*	104,3	12,1	*
		90	114,6	14,2	*	40,31	1,79	**	26,10	3,68	*	79,62	6,90	**
		30	61,75	13,1	*	236,6	50,1	*	95,26	21,5	*	135,2	25,6	*
	12 mg.kg^{-1}	60	4,18	1,28	*	55,26	14,1		51,93	15,1		29,53	7,04	*
		90	86,90	7,11		8,95	1,53	**	7,62	0,57	*	48,74	2,67	*
		30	0,69	0,09		0,85	0,08		0,73	0,10		0,75	0,09	
	0 mg.kg^{-1}	60	0,29	0,02		0,26	0,05		0,38	0,11		0,27	0,02	
		90	0,42	0,03		0,34	0,01		0,35	0,05		0,38	0,01	
		30	5,14	1,38		47,58	9,06	*	54,65	10,8	*	21,00	3,18	*
IMC60	6 mg.kg^{-1}	60	4,45	0,08	**	10,06	1,54	*	21,00	1,98	**	6,99	0,71	**
		90	6,46	0,12	**	18,28	2,02	**	8,61	0,93	**	12,68	1,06	**
		30	25,75	6,14	*	114,12	25,28		71,64	14,12		57,38	9,66	
	12 mg.kg^{-1}	60	10,51	1,16	*	30,86	6,25		39,36	9,29		19,88	3,29	
		90	53,67	8,10	*	78,07	5,60	*	19,92	3,57		65,88	3,06	**
		30	0,64	0,08		0,80	0,12		0,80	0,17		0,69	0,09	
	0 mg.kg^{-1}	60	0,45	0,07		0,31	0,06		0,28	0,02		0,40	0,02	
		90	0,35	0,03		0,28	0,04		0,27	0,03		0,32	0,03	
		30	4,99	1,25	*	21,84	4,35	*	78,79	12,87	*	10,34	1,99	*
IMC67	6 mg.kg^{-1}	60	3,25	0,29	**	6,44	0,67	**	36,21	2,07	**	4,68	0,43	**
		90	6,62	0,54	**	19,00	1,65	**	6,43	0,31	**	12,60	1,03	**
		30	7,70	1,40		22,08	2,56		95,70	17,73		12,91	1,60	
	12 mg.kg^{-1}	60	5,98	1,49		12,75	2,09	*	57,04	12,56		8,78	1,61	
		90	8,09	0,53		56,18	6,27	*	12,52	0,82	*	29,77	2,29	*
		30	0,82	0,18		1,54	0,50		1,12	0,25		1,07	0,20	
	0 mg.kg^{-1}	60	0,31	0,03		0,58	0,18		0,53	0,15		0,42	0,09	
		90	0,29	0,02		0,24	0,01		0,23	0,04		0,27	0,01	
		30	21,59	4,81	*	20,48	5,34	*	46,86	5,21	*	21,07	4,41	*
PA121	6 mg.kg^{-1}	60	5,03	0,46	**	7,81	1,13	*	62,44	7,67	*	6,26	0,75	*
		90	7,00	0,30	**	33,74	4,92	*	10,82	1,40	*	19,91	2,00	*
		30	8,15	0,90		23,15	4,65		166,43	17,46	*	13,11	1,85	
	12 mg.kg^{-1}	60	6,29	1,50		9,74	1,60		75,62	6,86		7,88	1,52	
		90	15,96	1,97	*	86,85	5,79	*	18,85	1,47	**	52,75	2,33	**
		30	0,47	0,05		0,75	0,10		0,59	0,03		0,58	0,05	
	0 mg.kg^{-1}	60	0,28	0,05		0,37	0,05		0,30	0,03		0,32	0,05	
		90	0,44	0,05		0,26	0,03		0,30	0,01		0,36	0,02	
		30	4,26	0,43	**	7,30	1,45	*	16,25	3,06	*	5,39	0,33	**
PA150	6 mg.kg^{-1}	60	21,90	1,85	**	8,51	1,62	*	12,88	3,07	*	15,23	1,12	**
		90	93,78	8,35	**	38,30	6,63	*	10,81	0,74	**	67,63	2,53	**
		30	4,64	0,45		7,88	1,31		29,81	3,32	*	5,97	0,61	
	12 mg.kg^{-1}	60	103,95	21,75	*	8,69	1,87		17,28	3,10	*	61,43	13,24	
		90	110,64	15,29		126,63	7,88	*	12,51	1,36		118,67	11,47	*
		30	0,69	0,17		0,69	0,10		0,84	0,17		0,68	0,14	
	Control	60	0,47	0,02		0,31	0,05		0,32	0,06		0,40	0,01	
		90	0,46	0,06		0,31	0,04		0,37	0,06		0,39	0,05	
		30	5,43	1,57		20,34	3,41	*	58,65	6,33	**	11,33	2,36	*
PA46	6 mg.Kg^{-1}	60	4,23	0,82	*	12,95	2,83	*	36,67	4,63	*	7,95	1,38	*
		90	7,20	1,25	*	25,49	2,94	**	9,58	1,22	*	15,78	1,79	**
		30	13,56	1,36	**	72,46	5,99	*	16,74	2,94	*	37,18	2,46	**
	12 mg.Kg^{-1}	60	14,33	2,10	*	50,70	8,11	*	16,83	2,28		30,40	5,03	*
		90	24,89	3,78	*	18,52	3,63		15,65	3,38		21,56	1,65	
		30	0,46	0,15		0,82	0,07		1,35	0,12		0,61	0,11	
	0 mg.kg^{-1}	60	0,56	0,12		0,36	0,04		0,24	0,01		0,47	0,08	
		90	0,41	0,06		0,49	0,07		0,37	0,04		0,45	0,05	
		30	8,37	0,45	**	22,31	2,05	**	113,60	10,43	**	13,92	1,04	**
SCC85	6 mg.kg^{-1}	60	4,13	0,66	*	6,97	0,99	*	46,01	6,68	*	5,43	0,16	**
		90	7,69	0,41	**	73,34	4,73	**	14,92	3,25	*	40,88	3,62	**
		30	7,45	0,31		28,49	3,27		136,07	11,65		15,22	1,54	
	12 mg.kg^{-1}	60	10,27	1,85	*	19,72	3,82		75,54	13,47		14,57	1,96	*
		90	14,79	1,46	*	131,13	6,64	**	23,17	3,49		74,51	6,00	*
		30	0,70	0,26		0,73	0,09		0,79	0,14		0,70	0,20	
	0 mg.kg^{-1}	60	0,60	0,17		0,50	0,14		0,49	0,08		0,55	0,15	
		90	0,58	0,04		0,32	0,07		0,41	0,07		0,44	0,06	
		30	5,09	1,12	*	14,89	0,65	**	95,16	7,70	**	8,68	0,61	**
SCC86	6 mg.kg^{-1}	60	3,89	0,69	*	6,84	1,09	*	26,58	1,95	**	5,28	0,89	*
		90	6,26	0,24	**	65,22	3,99	**	10,69	1,40	*	35,62	1,25	**
		30	9,41	2,06	*	27,32	1,72	*	122,78	9,74	**	15,79	1,79	*
	12 mg.kg^{-1}	60	8,21	1,15	*	14,89	0,87	**	97,89	11,39	*	10,92	1,15	**
		90	13,55	0,47	**	86,47	8,11		16,17	1,64	*	48,80	4,04	

[a] mg Cd kg^{-1} DW

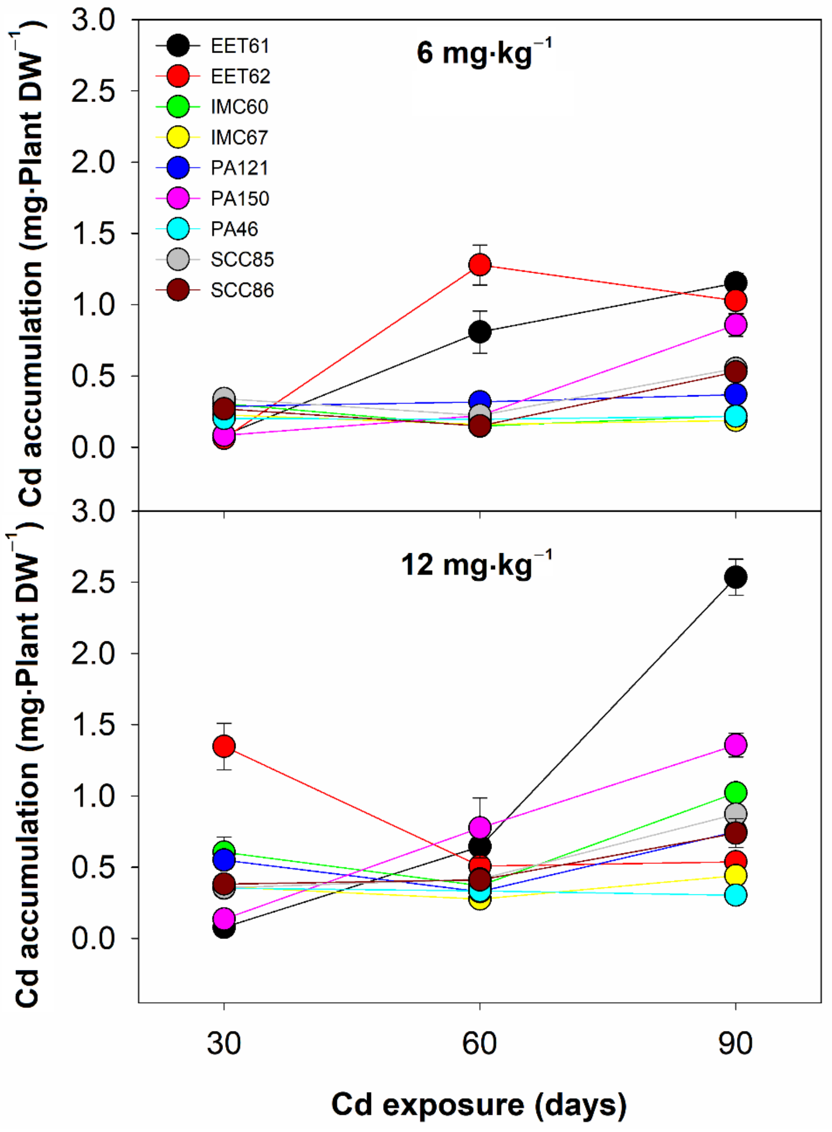

Figure S5. Total cadmium accumulated per plant in different genotypes of cacao rootstock over time. Values of total cadmium accumulated per plant are obtained from the sum of the accumulated values in each part of the plant (leaves, stems, and roots). The cadmium values allocated to each plant part are obtained through the relationship between concentration (mg.kg^{-1}) and dry mass (kg DW) of each plant part, respectively. Circles represent mean (n=3), and bars represent standard error of mean (SEM).
